# Antimicrobial Activity and Cytotoxicity of Ag(I) and Au(I) Pillarplexes

**DOI:** 10.3389/fchem.2018.00584

**Published:** 2018-11-27

**Authors:** Alexander Pöthig, Sara Ahmed, Hanne Cecilie Winther-Larsen, Shengyang Guan, Philipp J. Altmann, Jürgen Kudermann, Adriana Magalhães Santos Andresen, Tor Gjøen, Ove Alexander Høgmoen Åstrand

**Affiliations:** ^1^Department of Chemistry and Catalysis Research Center, Technical University of Munich, Garching, Germany; ^2^Department of Pharmaceutical Biosciences, School of Pharmacy, University of Oslo, Oslo, Norway; ^3^Department of Pharmaceutical Chemistry, School of Pharmacy, University of Oslo, Oslo, Norway

**Keywords:** NHC, silver, gold, antimicrobial, cytotoxicity, organometallic, supramolecular, stability

## Abstract

The biological activity of four pillarplex compounds featuring different metals and anions was investigated. The toxicity of the compounds against four bacterial strains [*Bacillus subtilis* (ATCC6633), *Staphylococcus aureus* (ATCC6538), *Escherichia coli* (UVI isolate), *Pseudomonas aeruginosa*], one fungus (*Candida albicans*), and a human cell line (HepG2) was determined. Additionally, a UV-Vis titration study of the pillarplexes was carried out to check for stability depending on pH- and chloride concentration changes and evaluate the applicability in physiological media. All compounds are bioactive: the silver compounds showed higher activity against bacteria and fungi, and the corresponding gold pillarplexes were less toxic against human cells.

## Introduction

**Figure 1:**
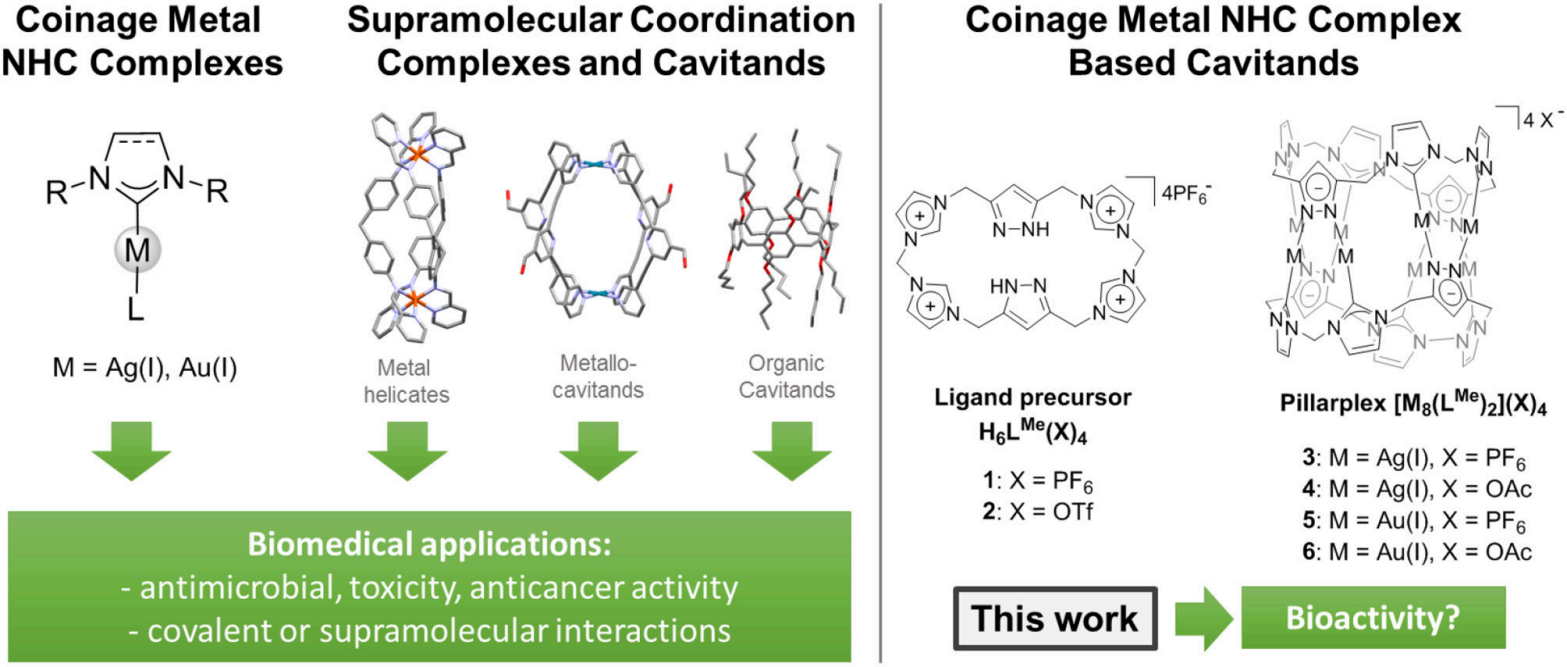
**(Left)** Overview of related known biomedical relevant compound classes. **(Right)** NHC based organometallocavitands including precursor investigated in this study.

Since the early 2000s, coinage metal complexes featuring N-heterocyclic carbenes (NHC)—a ligand class with a facile tunability toward sterics, electronics, and solubility—have been employed as bioactive compounds (Herrmann, 2002; Mercs and Albrecht, 2010; Hopkinson et al., 2014). As first examples, silver (I) NHC complexes have been used as antimicrobial compounds, pioneered by Youngs et al. (Kascatan-Nebioglu et al., 2004; Melaiye et al., 2004), and a respective applicability of such compounds has been shown for a variety of complexes ever since (Figure 1) (Kascatan-Nebioglu et al., 2007; Hindi et al., 2009; Oehninger et al., 2013; Liang et al., 2018). Hereby, a slow release of silver ions originating from the decomposition of the NHC complexes is expected to be the cause of their activity, which can be rationalized by the comparably labile metal-carbene bond (with respect to other late transition metal-NHC bonds) (Kascatan-Nebioglu et al., 2007). The more stable gold (I) NHC complexes were also employed in studies investigating their antibiotic potential (Lazreg and Cazin, 2014). One possible target are (seleno)-cysteine moieties in proteins, e.g., thioredoxin reductase, accompanied by the inhibition of the enzyme, which is similar to the mode of action proposed for the approved metallodrug Auranofin (Baker et al., 2005; Schuh et al., 2012). This is expected in particular for gold(I) mono-carbene complexes, which can dissociate one (labile non-NHC) ligand to coordinate the sulfur or selenium atom (Rubbiani et al., 2011, 2013; Cheng et al., 2014; Meyer et al., 2014; Arambula et al., 2016; Bertrand et al., 2017; Karaca et al., 2017a; Schmidt et al., 2017; Zhang et al., 2018). In case of the di-NHC complexes, which are more stable toward dissociation, a different mode of action can be observed. Casini and coworkers were able to show stacking of Au(I) di-caffeine NHC complexes in G4 quadruplex DNA structures, inhibiting telomerase activity (Bertrand et al., 2014; Bazzicalupi et al., 2016; Karaca et al., 2017b). Hereby, the overall structure of the intact complex (being planar, cationic, and possessing a conjugated system for stacking) determines the ability to interact in a non-covalent binding, forming supramolecular aggregates. A related supramolecular recognition of biomolecules causing bioactivity was discovered by Michael Hannon and coworkers, who were using cylindrical metal helicates—a class of supramolecular coordination complexes (SCCs, Figure 1)—to interact with different DNA structures (Meistermann et al., 2002; Oleksi et al., 2006; Hannon, 2007; Ducani et al., 2010; Phongtongpasuk et al., 2013; Malina et al., 2016). They showed, that the overall charge of the compounds (4+) as well as the aromatic parts of the ligands were crucial for supramolecular recognition of the negatively charged DNA. In general, such supramolecular coordination compounds are discussed as a promising class for future applications as metallodrugs or drug delivery systems (Casini et al., 2017).

We recently introduced the pillarplexes (Figure 1), a new family of SCCs which are structurally similar to Hannon's cylindrical helicates yet additionally exhibit a pore which allows for encapsulation of guest molecules inside the complex (Altmann and Pöthig, 2016). These compounds are octanuclear coinage metal complexes with two coordinating macrocyclic NHC ligands. Due to their in-built functionality (e.g., luminescence, easy tunable solubility) caused by the metal-complex character, the pillarplexes are even more versatile than their highly successful organic relatives—the pillararenes (Ogoshi et al., 2008, 2016). The latter have also been applied in biomedical applications very recently, for reducing cytotoxicity, and improving the anticancer bioactivity of oxaliplatin (Hao et al., 2018). In case of the pillararenes and metallocage systems (Casini et al., 2017), the cavitand itself shows no bioactivity and therefore can be used to modulate the selectivity and activity of an actual metallodrug.

Our pillarplexes combine the possibilities to behave like NHC complexes, i.e., as a metallodrug themselves, with the possible applications of cavitands. Therefore, to explore the future potential of our pillarplexes in the biomedical context, we conducted a toxicity study. We tested the antimicrobial activity of the four metal complexes (**3–6**), the metal salts as well as the ligand precursor salts (**1**, **2**) toward four different bacterial strains [*Bacillus subtilis* (ATCC6633), *Staphylococcus aureus* (ATCC6538), *Escherichia coli* (UVI isolate), *Pseudomonas aeruginosa*] as well as one fungus (*Candida albicans*). We also evaluated the toxicity of the complexes toward a human cell line (HepG2) in order to clarify if related future research directions might be promising to follow. Finally, we conducted a stability study of the pillarplexes toward changes in pH and chloride ion concentration, which has implications on the use of the compounds under physiological conditions.

## Experimental section

### General details

Compounds **1**–**6** were prepared according to the reported procedures (Altmann et al., 2015; Altmann and Pöthig, 2016). Chemicals were purchased from commercial suppliers and used without further purification if not stated otherwise. Liquid NMR spectra were recorded on a Bruker Avance DPX 400 and a Bruker DRX 400 at room temperature if not stated otherwise. Chemical shifts are given in parts per million (ppm) and the spectra were referenced by using the residual solvent shift as internal standards. Emission spectra were recorded on a Agilent Cary 60 UV-Vis. Nutrient agar plates were prepared according to the instructions provided by Oxoid where 28 g of nutrient agar (CM0003) was needed to make 1 L of nutrient agar broth. 11.2 g of the agar was added to three 400 ml glass bottles. Four hundred milliliters of distilled water was added into each bottle containing the nutrient agar and was dissolved by stirring. After sterilization, the nutrient agar bottles were cooled to 50°C and then placed into a 50°C water bath for the temperature to remain constant. The nutrient agar was then poured halfway into 9 cm sterile petri dishes in HEPA filtered laminar flow cabinets to minimize the risk of contamination. The nutrient agar plates were then left to solidify and were refrigerated at 4°C. Mueller-Hinton agar plates were prepared from Mueller-Hinton agar medium (Sigma-Aldrich) and agar (Oxoid LP0011). Twenty-two grams of the Mueller-Hinton medium was added into 1 L of distilled water in a volumetric flask and dissolved with a magnetic flea at speed 6–7 and temperature 300°C for ~10 min (IKA Labortechnik). Fifteen grams of agar was added to the mixture and the stirring continued at speed 5–6 and at temperatures between 200 and 250°C until the mixture began to boil. After sterilization, the Mueller-Hinton broth was cooled to 50°C and stirred slightly with a magnetic flea for ~1 min (IKA Labortechnik). Sixty milliliters of the Mueller-Hinton broth was poured into each 13 cm petri dish using the media dispensing machine (IBS Integra Biosciences Technomat) using aseptic techniques. The Mueller-Hinton plates were allowed to cool and then stored in a refrigerator at 4°C. Plates were sterilized and stored at 4°C in the refrigerator before use. One liter of a 0.9% solution of sodium chloride was prepared and sterilized at 121°C for 20 min at 1 atm. The bacteria were streaked onto a nutrient agar plate using a sterile loop and incubated at 37°C overnight. The fungus was streaked using a sterile loop onto a TSA plate and incubated at 25°C for 48 h. Fresh streaks were prepared for each disc diffusion assay.

### Disc diffusion assays for antimicrobial activity

Antimicrobial activity was measured using the disc diffusion assay essentially as described in guidelines from Clinical and Laboratory Standards Institute CLSI (2012). The bacteria were maintained on Nutrient agar (Oxoid), while the fungus was maintained on Sabouraud dextrose agar (Oxoid). An inoculum of the test microorganisms were made by resuspending freshly overnight grown colonies into 2 mL of a sterile salt solution (0.9% NaCl). The test organism was diluted to McFarland standard density no. 2 and mixing thoroughly (McFarland, 1907). For the Gram-negative bacteria and fungus, 60 μL of the inoculum was added to 25 mL of sterile salt solution, while 120 μL was added for the Gram-positive bacteria.

To prepare the plates for the disc diffusion assay Mueller-Hinton agar 2 (Sigma-Aldrich) were covered with 5 mL of the freshly made inoculate. The surplus inoculate was removed and the plates were then left in a laminar flow hood until the surface of the plates were completely dry.

Six millimeter filter discs were impregnated with 10 μL volume of the ligand precursor compounds **1** [L(PF_6_)_4_] and **2** [L(OTf)_4_], silver pillarplexes **3** [Ag_8_L_2_(PF_6_)_4_] and **4** [Ag_8_L_2_(OAc)_4_], and gold pillarplexes **5** [Au_8_L_2_(PF_6_)_4_] and **6** [Au_8_L_2_(OAc)_4_]. The concentration of the compounds used were 10 mM. Further filter discs were also impregnated with 10 μl of: dimethyl sulfoxide (DMSO) acting as a negative control; 10 mM of silver nitrate, 10 mM of gold chloride acting as model compounds for free metal ions; and antibiotic discs including pre-impregnated 30 mg/ml gentamycin sulfate discs (BD BBL Sensi-Disk) (*E. coli, S. aureus, P. aeruginosa*), pre-impregnated 30 mg/ml tetracycline discs (BD Sensi-Disc) (*B. subtilis*), and 10 mM of Miconazole nitrate discs (Sigma-Aldrich) (*C. albicans*), acting as positive controls. The filter discs were placed evenly on 13 cm Mueller-Hinton agar plates separated to avoid overlapping inhibitions zones. The plates were incubated overnight at 32°C for the bacteria or 25°C for the fungus. The inhibition zones were measured with a caliper. All experiment was performed at least three times.

### *In vitro* toxicity in HepG2 liver cells

Human hepatocarcinoma cell line HepG2 (HB-8065, ATCC, Manassas, VA, USA) was cultured in MEM-Glutamax (5.5 mM glucose) supplemented with 10% fetal bovine serum (Gibco, Life Technologies AG, Basel, Switzerland), 100 μg/mL streptomycin, and 100 units/mL penicillin (both from Gibco, Life Technologies AG, Basle, Switzerland). Cells were incubated at 37°C under a 5% CO_2_ atmosphere. For viability assays, cells were seeded in white 96-well Nunc plates at a density of 20,000 cells/well and left overnight to adhere before experiments were conducted.

The compounds were dissolved in DMSO at concentrations ranging from 10^−3^ to 10^−6^ M and were added to white 96-well plates (maximum DMSO concentration in wells was lower than 1%) containing 20,000 HepG2 cells/well. Plates were incubated for 24 h at 37°C in a 5% CO_2_ atmosphere. After 24 h, AlamarBlue cell viability reagent (Thermo Fisher, Carlsbad, CA, USA) was added as a 10% solution, and plates were placed back in the incubator for 4 h. AlamarBlue is a redox indicator yielding a fluorescence signal proportional to the number of viable cells in each well (O'Brien et al., 2003). The fluorescence signal was measured in a microplate reader (Clariostar, BMG Labtech, Ortenberg, Germany) at 550 nm/603 nm (excitation/ emission). Data from four replicates were used to calculate the half-maximal inhibitory concentration (IC_50_) using Sigmoidal, 4PL, where X is log(concentration) analysis, and a four-parameter logistic regression from GraphPad Prism 7 (GraphPad Software Inc., USA). The experiment was repeated twice with similar results.

### UV-Vis experiments

#### Stability tests of pillarplexes against chloride

The titrations of the silver pillarplex **4** and gold pillarplex **6** against chloride ions were carried out by stepwise addition of an increasing volume of a 3.072 M sodium chloride solution to 2 mL of a 1.38 · 10^−5^ M aqueous pillarplex solution followed by thorough mixing in a quartz cuvette. The UV-Vis absorption spectra were recorded immediately after the addition. The measured absorbance was corrected for the increase of the sample volume.

#### Stability tests of pillarplexes against pH

The stability of silver pillarplex **4** and gold pillarplexes **6** in different concentrations of trifluoromethanesulfonic acid (HOTf) was monitored by UV-Vis spectroscopy. One milliliter of a 2.76 · 10^−5^ M aqueous pillarplex solution were injected into an equal volume of 1 mL HOTf solution with pH-values 2, 4, 5, and 6 in the quartz cuvette. The absorption spectra were recorded after 1 min, 1, 7, 24, 48, and 72 h (see Supplementary Material).

## Results and discussion

### Antimicrobial activity studies

The results of the antimicrobial studies are summarized in Table 1.

**Table 1:** Results from the disc diffusion assay for compounds **1**–**8** (10 mM) as well as reference substrates (30 mg/ml for tetracycline and gentamycine sulfate, and 10 mM for miconazole nitrate).

**Entry**	**Compound**	**mm no growth zone**				

		***E. coli***	***P. aeruginosa***	***S. aureus***	***B. subtilis***	***C. albicans***
1	H_6_L^Me^(PF_6_)_4_ (**1**)	0 ± 0	0 ± 0	0 ± 0	6.4 ± 0.4	0 ± 0
2	H_6_L^Me^(OTf)_4_ (**2**)	0 ± 0	0 ± 0	6.7 ± 0.4	6.8 ± 0.3	0 ± 0
3	[Ag_8_(L^Me^)_2_] (PF_6_)_4_ (**3**)	8.6 ± 0.2	8.0 ± 0.7	9.1 ± 1.7	8.3 ± 0.3	10.1 ± 0.4
4	[Ag_8_(L^Me^)_2_] (OAc)_4_ (**4**)	8.0 ± 0	8.2 ± 0.6	9.2 ± 2.1	7.8 ± 0.4	9.2 ± 0.9
5	[Au_8_(L^Me^)_2_] (PF_6_)_4_ (**5**)	7.4 ± 0.4	0 ± 0	7.4 ± 0.6	0 ± 0	0 ± 0
6	[Au_8_(L^Me^)_2_] (OAc)_4_ (**6**)	0 ± 0	0 ± 0	0 ± 0	0 ± 0	0 ± 0
7	AgNO_3_	7.8 ± 0.3	7.4 ± 0.6	8.9 ± 1.8	6.3 ± 0.5	9.6 ± 0.4
8	AuCl_3_	8.6 ± 0.4	8 ± 0	8.9 ± 1.7	7.3 ± 0.4	0 ± 0
9	DMSO^*^	0 ± 0	0 ± 0	0 ± 0	0 ± 0	0 ± 0
10	Miconazole nitrate^**^	–	–	–	–	17.1 ± 0.8
11	Tetracycline^**^	–	–	–	28.9 ± 0.5	–
12	Gentamycin sulfate^**^	23.7 ± 1.6	23.0 ± 0.7	23.7 ± 1.6	–	–

* *negative control*,

** *positive control*.

Both silver compounds (entries 3 and 4) show antimicrobial activity against all bacterial strains as well as the fungus. Hereby, the activity is independent of the anion present, as the results are identical within the margin of errors. In comparison to the positive controls (entries 10–12) the overall activity is moderate, however, (by means of statistic uncertainty) it is identical to that of AgNO_3_ (entry 7), which has been used as an antibiotic since ancient times (Danscher and Locht, 2010). Hence we suspect the release of silver ions via decomposition of the pillarplexes, which is in agreement with the general behavior of silver(I) NHC complexes, as stated above.

The gold pillarplexes show lower to no activity (entries 5 and 6). Compound **6**, the completely water soluble acetate, shows no activity against any of the microbes, whereas, the more lipophilic compound **5** shows a selective moderate activity against Gram-negative *E. coli* and Gram-positive *S. aureus*. In contrast, AuCl_3_ shows activity against all bacterial strains (interestingly not against the fungus), which of course might be additionally influenced by the redox activity of the gold(III) ion. However, we suspect the gold pillarplexes being more stable in the physiological environment, therefore not releasing uncoordinated metal ions, which would explain the lower activity. Similarly, if the gold complexes would decompose, a similar toxicity as in case of the free ligand precursors would be expected. In general, such imidazolium salts are known to be potentially toxic, depending on different factors, e.g., lipophilicity or anions (Gravel and Schmitzer, 2017). In our case, the two macrocyclic polyimidazolium ligand precursors (entries 1 and 2) show only moderate and very selective toxicity only against the Gram-positive bacteria *S. aureus* and *B. subtilis*. Gram-positive bacteria lacks the outer membrane surrounding the cell wall. This outer membrane excludes, by various mechanisms, certain drugs from penetrating the bacterial cell (Hancock, 1997) and could be the reason for antimicrobial selectivity of compound **2**. For the latter, no activity at all was observed in case of the gold pillarplexes, why we rule out a possible decomposition.

### Cell toxicity studies

The results for the toxicity study of the compounds against human HepG2 liver cells are summarized in Table 2. The IC_50_-values were determined for all compounds, however, the silver pillarplexes (3 and 4) as well as AgNO_3_ and AuCl_3_ all showed precipitation to some degree. This can influence both uptake of the compounds by the cells and the absorbance read, resulting in ambiguous measurement results, which we have pointed out by an asterisk in Table 2.

**Table 2:** IC_50_-values for compounds **1**–**6** as well as reference salts against human liver cells (HepG2).

**Compound**	**1**	**2**	**3**	**4**	**5**	**6**	**AgNO_3_**	**AuCl_3_ 3H_2_O**
IC_50_ ± SE (μM)	467.5 ± 1.4	402.2 ± 1.1	88^*^	56^*^	72.4 ± 1.0	29.9 ± 1.0	127^*^	274^*^

* *Precipitation occurred, therefore IC_50_ results are ambiguous and no SE could be estimated for these compounds (see Supplementary Material)*.

In general, all tested compounds exhibit biological activity. Both ligand precursors (**1** and **2**) exhibited low toxicity levels which corresponds to the determined IC_50−_values. In contrast, high cell toxicity was observed in concentration higher than 100 μM for all pillarplexes (see figures in the Supplementary Material). According to the determined IC_50_-values, the gold congeners are more active within the pairs of pillarplexes with the same anions (**3** vs. **5** and **4** vs. **6**). However, they also show a higher base RFU compared to the silver compounds, indicating the gold compounds to be less toxic. With regard to the effect of the anions, the more water-soluble compounds (**2**, **4**, **6**: triflates or acetates) show higher activity than the less water-soluble hexafluorophosphate salts (**1**, **3**, **5**). In general, the same trend as in the antimicrobial assay are observed with the HepG2 cells. The silver pillarplexes appear to be more toxic and more active than their gold counterparts. Precipitation was observed in case of the silver pillarplexes as well as for AgNO_3_ and AuCl_3_, whereas the gold pillarplexes did not exhibit any stability or solubility issues.

### Stability tests

**Figure 2:**
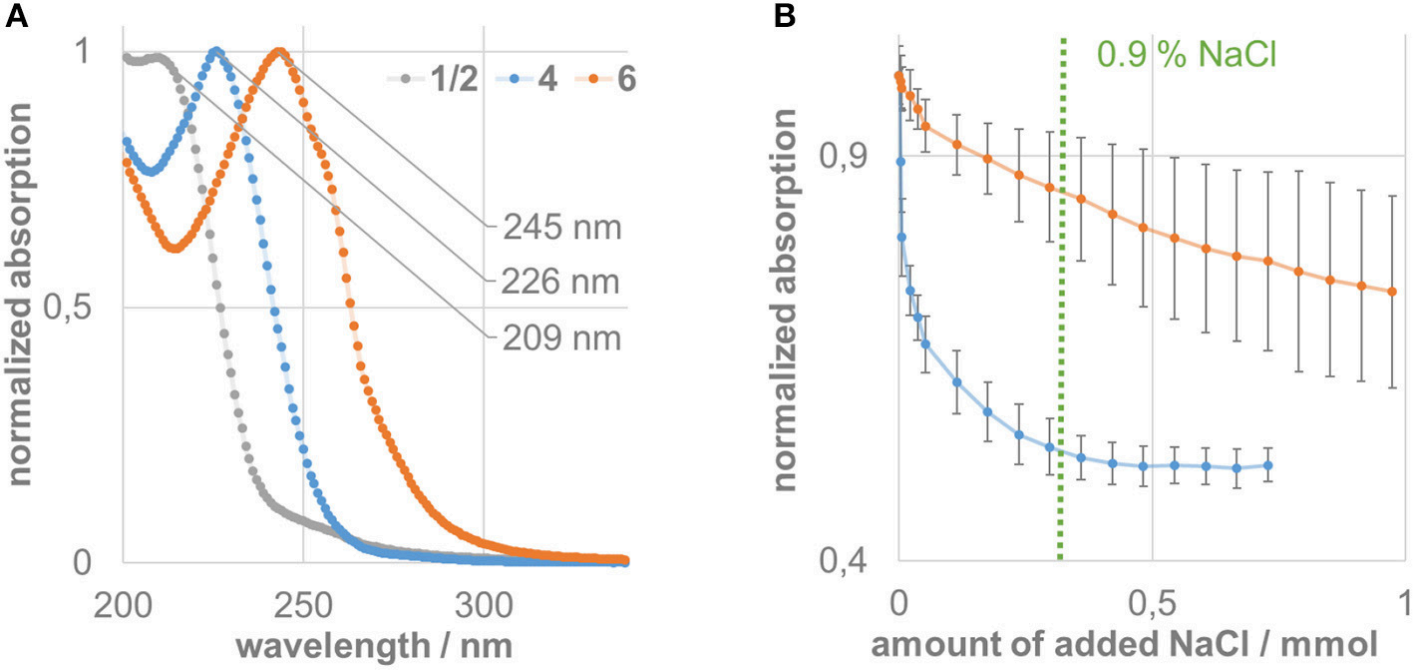
**(A)** Normalized UV-Vis absorption spectra of the ligand precursor (gray) and pillarplexes **4** (blue), and **6** (orange). **(B)** Titration experiment of compounds **4** (blue) and **6** (orange) against sodium chloride, showing the decay of the normalized signal at the individual absorption maximum (226 nm for **4**, 245 nm for **6**) including statistical uncertainties.

To evaluate possible reasons for the observations made during the bacterial and cell tests, we conducted an UV-Vis titration study. In detail, we checked the influence of a varying chloride and proton concentration on the stability or solubility of the pillarplex compounds. Therefore, we first evaluated the absorption properties of the two water-soluble pillarplex acetates **4** and **6** in aqueous solution, as well as the ligand precursor (Figure 2A). All compounds absorb in the UV range: the silver complex **4** shows an absorption maximum at 226 nm whereas the gold complex **6** absorbs at 245 nm. The ligand precursor absorbs at 209 nm. The molar extinction coefficients for the pillarplex compounds at the wavelengths of the individual maximal absorption are 9.33 · 10^4^ ± 6.41 · 10^2^ M^−1^cm^−1^ (**4**) and 1.21 · 10^5^ ± 4.64 · 10^3^ M^−1^cm^−1^ (**6**).

The titration results of the pillarplexes against an increasing amount of chloride ions present in aqueous solution show a very different behavior of the silver compared to the gold compound (Figure 2B). The absorption signal of silver complex **4** immediately drops up to addition of 0.5 mmol NaCl (which is about 17,000-fold excess of chloride). After that, no significant change can be observed in the absorption spectra upon addition of more equivalents of chloride. Apparently, this is close to the physiological concentration of chloride (0.9%) which might be a possible explanation why precipitation was observed in the biological tests for the silver containing pillarplexes. The gold compound **6** also shows a decay if the absorption signal upon chloride addition. However, the drop is less pronounced and at 0.9% chloride concentration, there is still a significant absorption (85% of the initial value). At higher chloride contents we observed a higher variation of the measured values, which we cannot explain up to now. However, even after addition of 1 mmol NaCl (about 35,000-fold excess) the characteristic absorption band at 245 nm can be observed for compound **6** (see Supplementary Material), strongly indicating that the gold complex is significantly less effected by chloride addition and still present in solution under physiological conditions.

**Figure 3:**
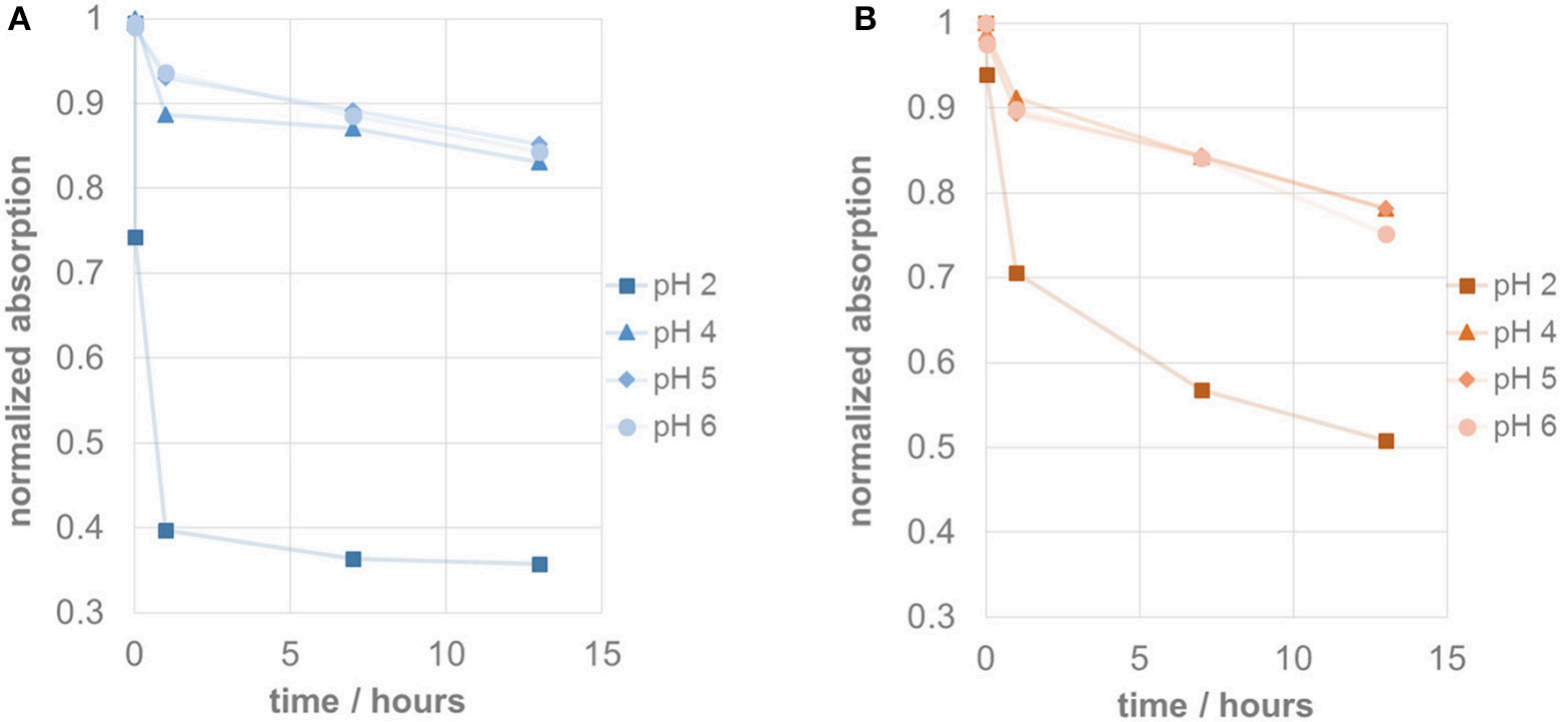
**(A)** Stability test at different pH-values showing decay of normalized absorption of silver pillarplex **4** (blue) at 226 nm over time. **(B)** Stability test at different pH-values showing decay of normalized absorption of gold pillarplex **6** (orange) at 245 nm over time.

Figure 3 shows the pH-dependent decay of pillarplex compounds **4** and **6** over time. From our previous work on pillarplex rotaxanes we already knew, that in case of silver, the metal ions can be released quickly in the presence of an excess of the strong trifluoromethanesulfonic acid (Altmann and Pöthig, 2017). This was reproduced also in case of the empty pillarplex **4** (Figure 3A) for which an immediate drop of absorption signal at 226 nm was observed at pH 2, indicating very fast decomposition to the protonated imidazolium precursor. The resulting UV-Vis spectrum is also in agreement to that measured for the ligand precursor (Figure 2A). At higher pH-values, the decomposition of **4** is significantly slower and almost identical for pH 4–6. A similar behavior was observed for the gold complex **6** although the decay at pH 2 is significantly slower than that of its silver analog (Figure 3B). Interestingly, at the higher pH-values the relative drop of the absorption signal is more pronounced compared to the silver complex. However, in case of **6** the resulting absorption spectrum after the assumed decomposition is not resembling that of the ligand precursor, and rather corresponds to the spectrum of **6** just with lower absorption intensity. Therefore, we additionally conducted a NMR experiment to check for protonation of the NHC ligands at pH 2. As a result, no protonated species was detected strongly indicating that the gold pillarplexes are stable even at low pH (see Supplementary Information Figure S15).

## Conclusion

In general, the silver pillarplexes behave like similar silver complexes and show antimicrobial and antifungal activity as well as moderate toxicity toward human HepG2 cells. The corresponding gold complexes were inactive against most bacterial strains and fungi, as well as had lower HepG2 toxicity. The observed effects originate most likely from the increased stability of the gold pillarplexes compared to the silver pillarplexes, as evident by the UV-Vis titration and the ^1^H NMR experiment. The fact that the gold complexes seem comparably non-toxic and stable opens up the possibility of them being used as drug carriers for selective drug delivery or modified release of drugs that could fit inside the cavity in the pillarplexes.

## Author contributions

AP: project conception and supervision, manuscript composition, and writing. PA: synthesis and characterization of pillarplexes. SG: synthesis and characterization of pillarplexes, UV-Vis studies. JK: UV-Vis studies. OH: biological testing supervision, data analysis, manuscript writing. SA biological testing, data analysis. HWL: biological testing, supervision, data analysis. AS: biological testing, data analysis. TG: biological testing supervision, data analysis.

### Conflict of interest statement

The authors declare that the research was conducted in the absence of any commercial or financial relationships that could be construed as a potential conflict of interest.

## Supplementary material

Additional information on the IC_50_ determinations as well as the UV-Vis experiments is available as supplementary material.
