# Supplementary material for: Antimicrobial Activity and Cytotoxicity of Ag(I) and Au(I) Pillarplexes
Source: Front Chem. 2018 Nov 27;6:584. doi: 10.3389/fchem.2018.00584 (PMC6277803; doi:10.3389/fchem.2018.00584)
Supplement: Supplementary file 1 [file Data_Sheet_1.PDF]

Supplementary Material

Antimicrobial Activity and Cytotoxicity of Ag(I) and Au(I) Pillarplexes

Alexander Pöthig[1*], Sara Ahmed[2], Hanne Cecilie Winther-Larsen[2], Shengyang Guan[1], Philipp, J. Altmann[1], Jürgen Kudermann,[1] Adriana Magalhães Santos Andresen[2], Tor Gjøen[2], and Ove Alexander Høgmoen Åstrand[3*]

[1] Department of Chemistry and Catalysis Research Center, Technical University of Munich, Lichtenbergstr. 4, 85748 Garching, Germany.

[2] Department of Pharmaceutical Biosciences, School of Pharmacy, University of Oslo, PO Box 1068 Blindern, 0316 Oslo, Norway

[3] Department of Pharmaceutical Chemistry, School of Pharmacy, University of Oslo, PO Box 1068 Blindern, 0316 Oslo, Norway.

*** Correspondence: Corresponding Author**
alexander.poethig@tum.de; o.a.h.astrand@farmasi.uio.no

Content

1 IC_{50} determinations against HepG2 cells 2
2 Stability study against addition of chloride ions 3
2.1 $Ag_8L_2(OAc)_4$ (4) against chloride 3
2.2 $Au_8L_2(OAc)_4$ (6) against chloride 5
3 Stability study against different pH 8
3.1 $Ag_8L_2(OAc)_4$ against pH 8
3.2 $Au_8L_2(OAc)_4$ against pH 9
4 ^1H NMR spectrum of $[Au_8L_2](OAc)_4$ (6) in D_2O at pH 2 15

1 IC_{50} determinations against HepG2 cells

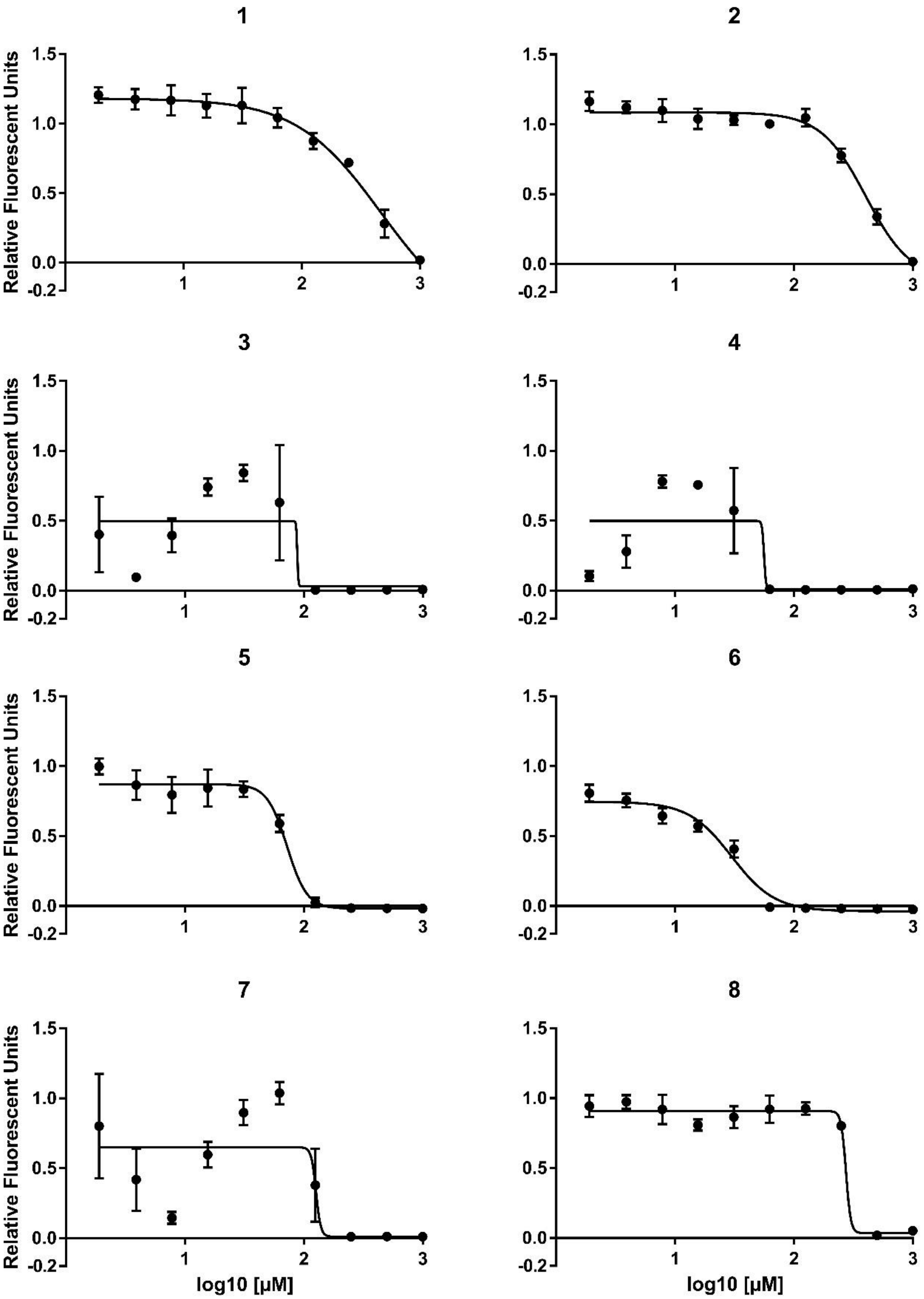

2 Stability study against addition of chloride ions

To evaluate the influence of a varying chloride concentration on the stability or solubility of the pillarplex compounds, an UV-Vis titration study was conducted. Stock solutions ($1.38 \cdot 10^{-5}$ M) of $Ag_8L_2(OAc)_4$ or $Au_8L_2(OAc)_4$ were prepared respectively, titrations were performed by adding increasing volume of 3.072 M sodium chloride solution to 2 mL of $1.38 \cdot 10^{-5}$ M pillarplexes water solution with thorough mixing in a quartz cuvette followed by UV-Vis spectroscopy. For each experiment, seventeen measurements were performed with different chloride concentrations. Titrations were repeated three times.

2.1 $Ag_8L_2(OAc)_4$ (4) against chloride

The characteristic absorption maximum of $Ag_8L_2(OAc)_4$ is located at 226 nm. By increasing the amount of chloride in solution, the absorption of pillarplex was decreasing rapidly. The absorption change in its UV-Vis spectra was listed in Table S1. The titration curve, shown in Figure S1, was obtained plotting the mean absorption at 226 nm with error bar against the amount of NaCl added.

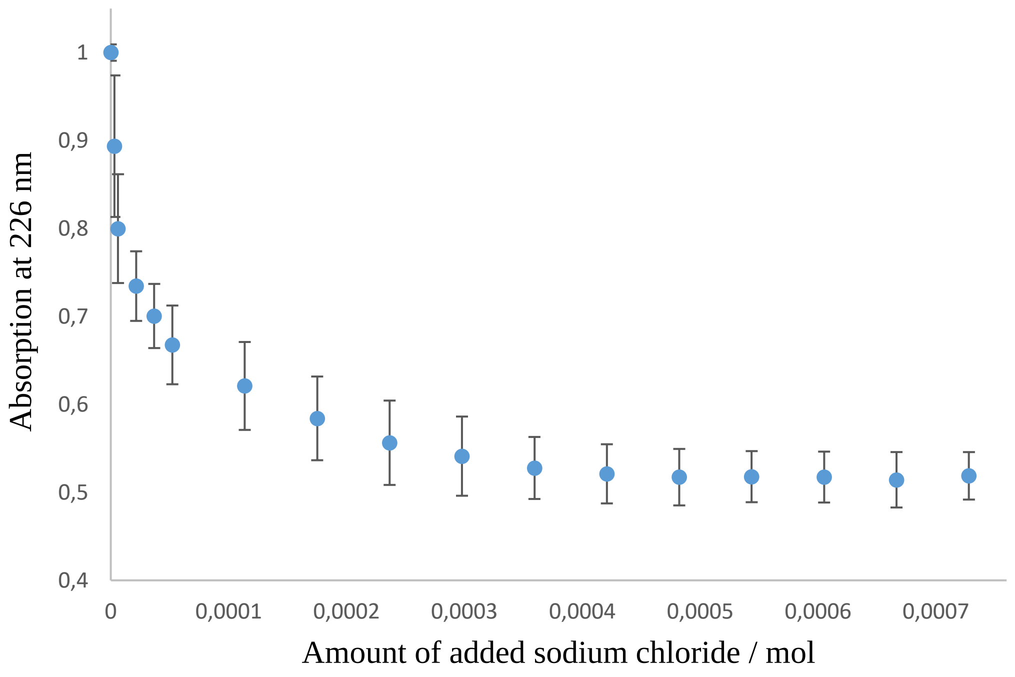

Figure S1: Plot of absorption at 226 nm of $Ag_8L_2(OAc)_4$ (dilution factor corrected) with NaCl in water showing a rapid decreasing since the addition of chloride.

Table S1: Data for the titration of $Ag_8L_2(OAc)_4$ and NaCl in water.

Number	Volume of brine added	mol of NaCl added	Absorption at 226 nm test 1	Absorption at 226 nm test 2	Absorption at 226 nm test 3
	mL	mol			
1	0	0	1.289	1.304	1.287
2	0.001	3.07E-06	1.072	1.232	1.1619
3	0.002	6.14E-06	0.963	1.076	1.064
4	0.007	2.15E-05	0.904	0.972	0.973
5	0.012	3.69E-05	0.864	0.922	0.931
6	0.017	5.22E-05	0.814	0.874	0.902
7	0.037	1.14E-04	0.748	0.817	0.844
8	0.057	1.75E-04	0.701	0.774	0.791
9	0.077	2.37E-04	0.667	0.731	0.761
10	0.097	2.98E-04	0.649	0.715	0.736
11	0.117	3.59E-04	0.643	0.694	0.711
12	0.137	4.21E-04	0.638	0.679	0.705
13	0.157	4.82E-04	0.634	0.675	0.698
14	0.177	5.44E-04	0.637	0.681	0.692
15	0.197	6.05E-04	0.636	0.682	0.689
16	0.217	6.67E-04	0.631	0.671	0.693
17	0.237	7.28E-04	0.641	0.677	0.694

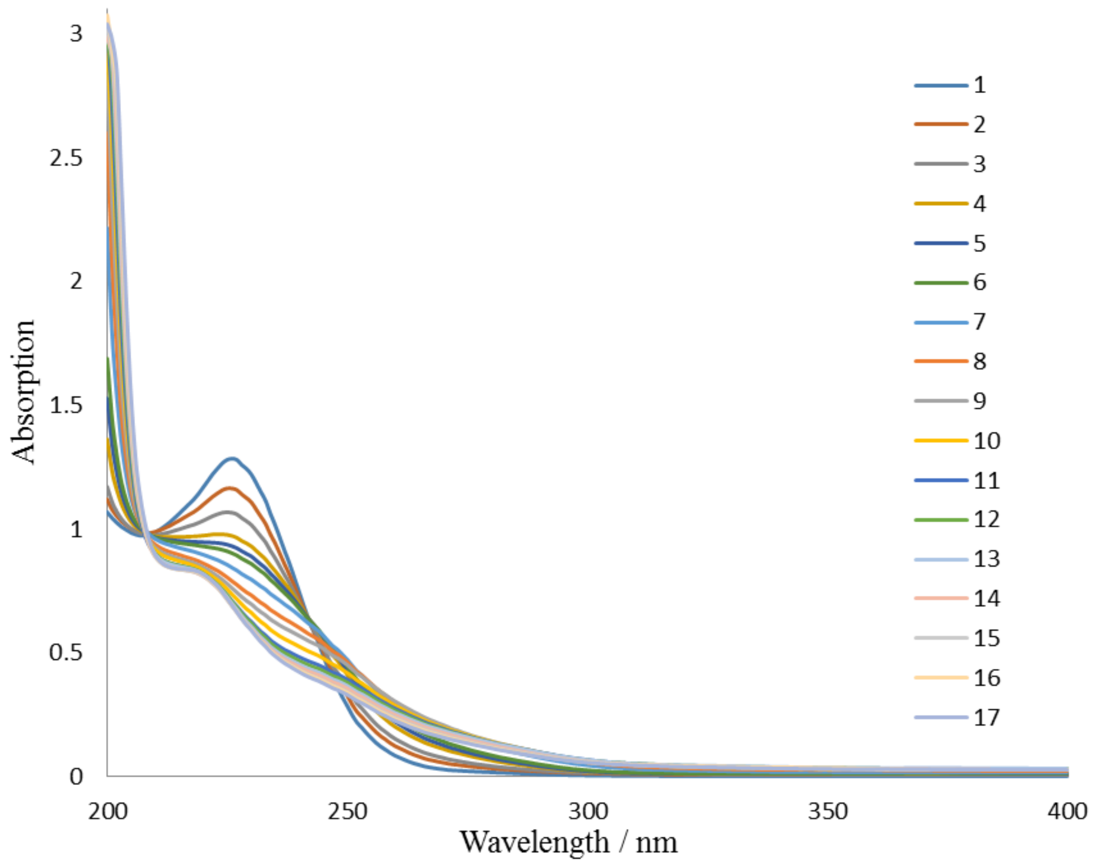

Figure S2: Stacked UV-Vis spectra of $Ag_8L_2(OAc)_4$ with an increasing mole amount of NaCl in water. The numbers on the right correspond to those in Table 1.

2.2 $Au_8L_2(OAc)_4$ (6) against chloride

The characteristic absorption maximum of $Au_8L_2(OAc)_4$ is located at 245 nm. By increasing the amount of chloride in solution, the absorption of pillarplex was decreasing gradually. The absorption change in its UV-Vis spectra is listed in Table S2. The titration curve, shown in Figure S3, was obtained plotting the mean absorption at 245 nm with error bar against the amount of NaCl added.

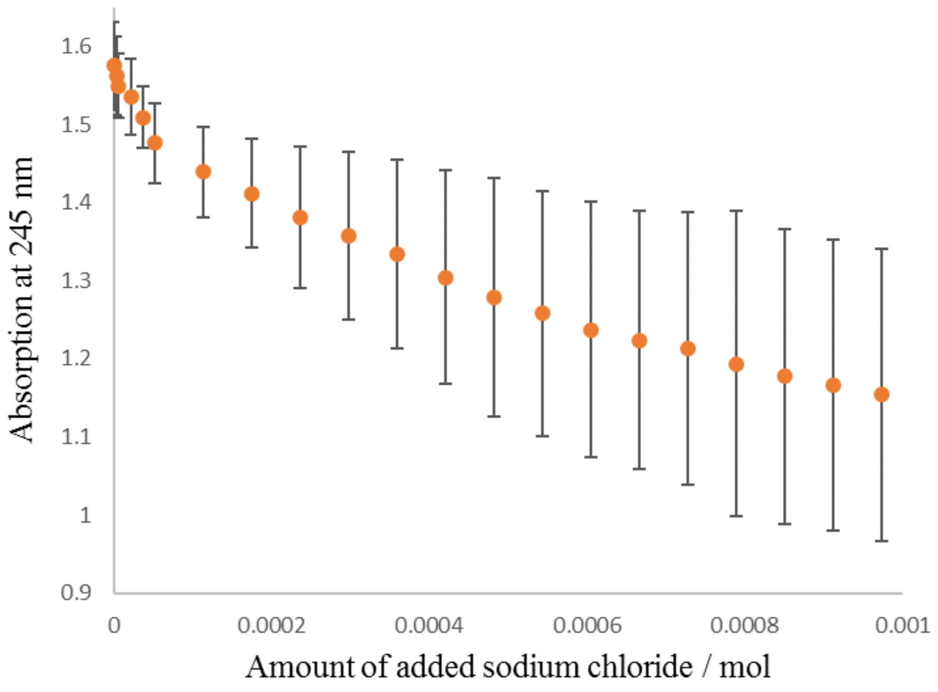

Figure S3: Plot of absorption at 245 nm of $Au_8L_2(OAc)_4$ (6) (dilution factor corrected) with NaCl in water showing a gradual decreasing since the addition of chloride.

Table S2: Data for the titration of $Au_8L_2(OAc)_4$ (6) and NaCl in water.

Number	Volume of brine added	mol of NaCl added	Absorption at 245 nm test 1	Absorption at 245 nm test 2	Absorption at 245 nm test 3
	mL	mol			
1	0	0	1.564	1.561	1.521
2	0.001	3.07E-06	1.559	1.551	1.507
3	0.002	6.14E-06	1.553	1.544	1.497
4	0.007	2.15E-05	1.537	1.534	1.466
5	0.012	3.69E-05	1.520	1.519	1.442
6	0.017	5.22E-05	1.498	1.489	1.389
7	0.037	1.14E-04	1.455	1.463	1.340
8	0.057	1.75E-04	1.424	1.437	1.307

9	0.077	2.37E-04	1.391	1.417	1.260
10	0.097	2.98E-04	1.365	1.397	1.223
11	0.117	3.59E-04	1.339	1.375	1.182
12	0.137	4.21E-04	1.317	1.343	1.129
13	0.157	4.82E-04	1.292	1.322	1.087
14	0.177	5.44E-04	1.265	1.293	1.062
15	0.197	6.05E-04	1.238	1.266	1.039
16	0.217	6.67E-04	1.215	1.245	1.017
17	0.237	7.28E-04	1.192	1.235	1.008
18	0.257	7.90E-04	1.172	1.226	0.983
19	0.277	8.51E-04	1.152	1.190	0.958
20	0.297	9.12E-04	1.133	1.167	0.932
21	0.317	9.74E-04	1.115	1.145	0.916

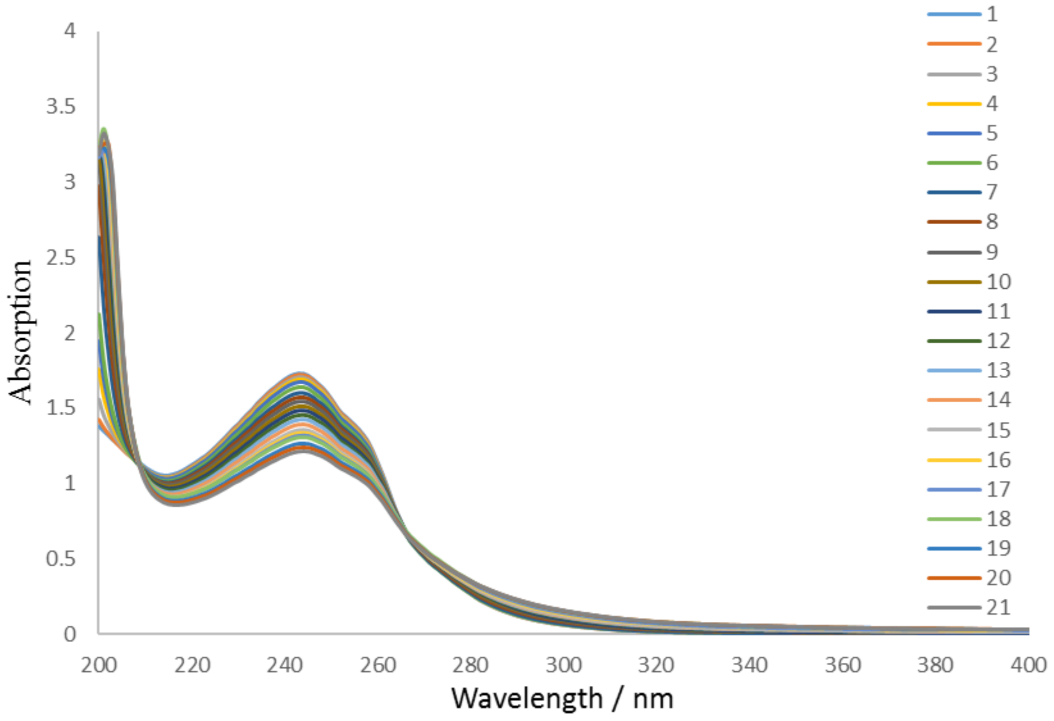

Figure S4: Stacked UV-Vis spectra of $Au_8L_2(OAc)_4$ with an increasing mole amount of NaCl in water. The numbers on the right correspond to those in Table 2.

3 Stability study against different pH

To evaluate the stability & solubility of the pillarplex compounds at different pH values, an UV-Vis study was conducted. Stock solutions ($2.76 \cdot 10^{-5}$ M) of $Ag_8L_2(OAc)_4$ or $Au_8L_2(OAc)_4$ were prepared respectively, titrations were performed by adding 1 mL of $2.76 \cdot 10^{-5}$ M pillarplexes solution to equal volume of HOTf water solutions at different pH with thorough mixing in a quartz cuvette followed by UV-Vis spectroscopy. For each experiment, measurements were performed after 1 min, 1 hour, 7 hours, 24 hours, 48 hours and 72 hours.

3.1 $Ag_8L_2(OAc)_4$ (4) against pH

The absorption change in its UV-Vis spectra was listed in Table S3. The decay curve, shown in Figure S5, was obtained plotting the absorption at 226 nm against time.

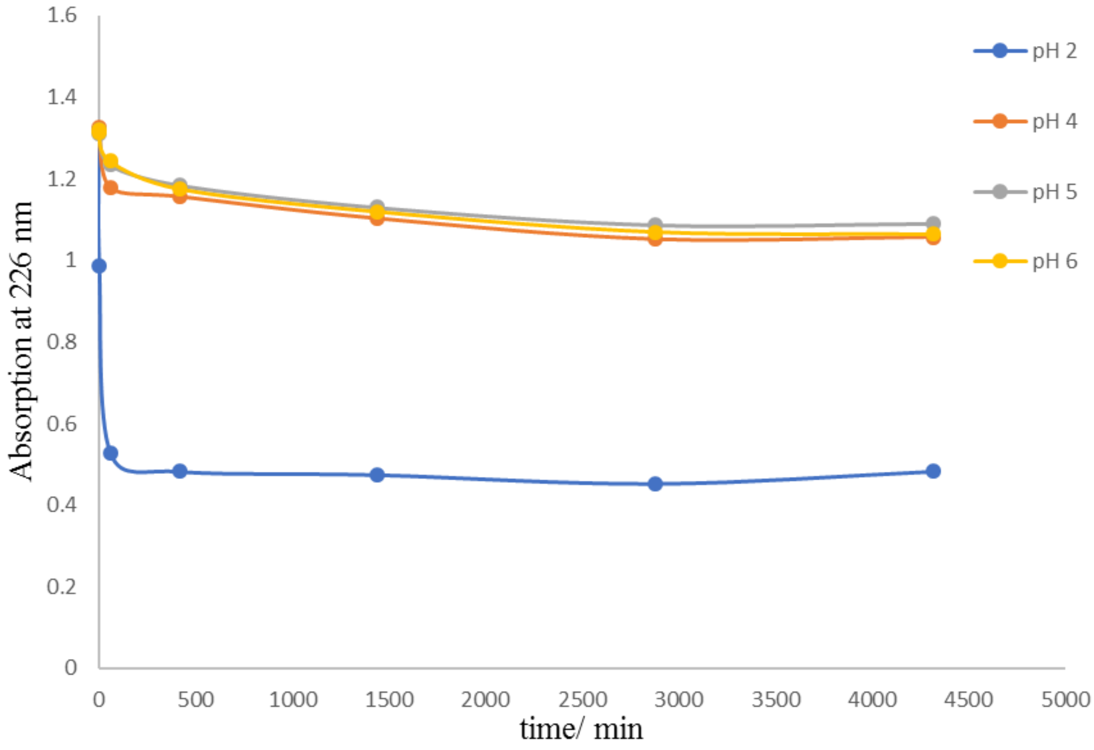

Figure S5: Plot of absorption at 226 nm of $Ag_8L_2(OAc)_4$ at different pH with time in water.

Table S3: Data for the decay of $Ag_8L_2(OAc)_4$ at different pH.

Time /mins	0	1	60	420	1440	2880	4320
Absorption at 226 nm at pH 2	1.319	0.986	0.527	0.482	0.474	0.453	0.483
Absorption at	1.319	1.326	1.177	1.155	1.102	1.051	1.056

226 nm at pH 4							
Absorption at 226 nm at pH 5	1.319	1.311	1.233	1.183	1.129	1.086	1.090
Absorption at 226 nm at pH 6	1.319	1.314	1.243	1.175	1.119	1.069	1.064

3.2 $Au_8L_2(OAc)_4$ (6) against pH

The absorption change in its UV-Vis spectra is listed in Table 4. The decay curve, shown in Figure S6 is obtained plotting the absorption at 245 nm against time.

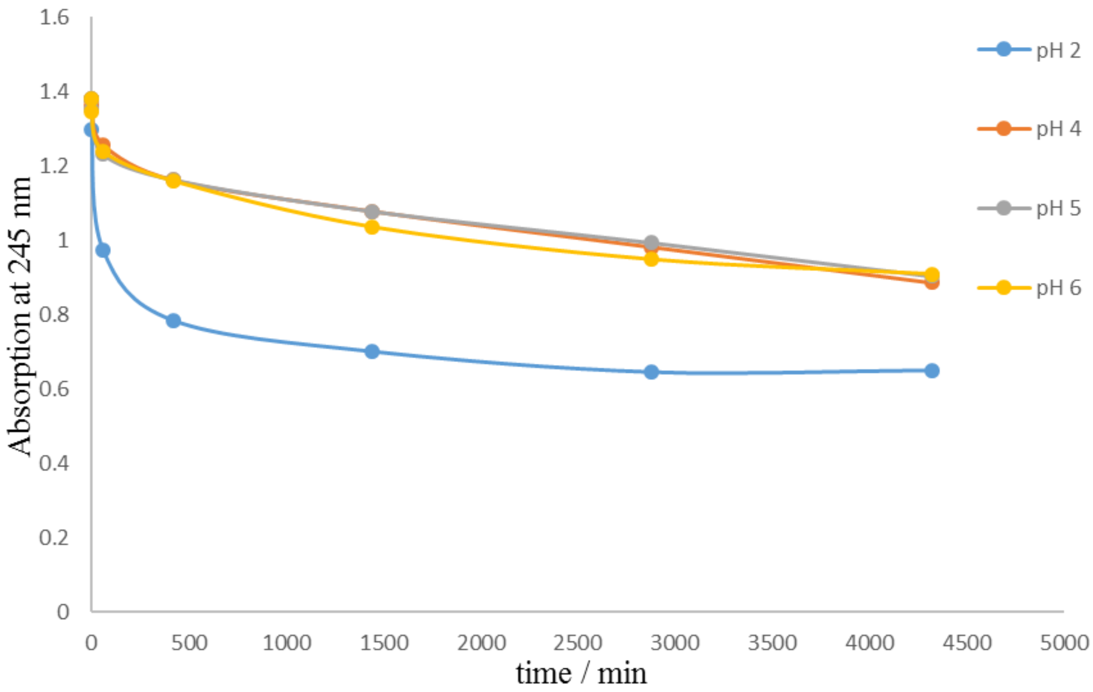

Figure S6: Plot of absorption at 245 nm of $Au_8L_2(OAc)_4$ at different pH with time in water.

Table S4: Data for the decay of $Au_8L_2(OAc)_4$ at different pH.

time/ min	0	1	60	420	1440	2880	4320
Absorption at 245 nm at pH 2	1.378	1.295	0.972	0.783	0.699	0.644	0.649
Absorption at 245 nm at pH 4	1.378	1.361	1.256	1.162	1.078	0.982	0.885
Absorption at 245 nm at pH 5	1.378	1.353	1.231	1.162	1.078	0.993	0.903
Absorption at 245 nm at pH 6	1.378	1.344	1.239	1.159	1.035	0.947	0.908

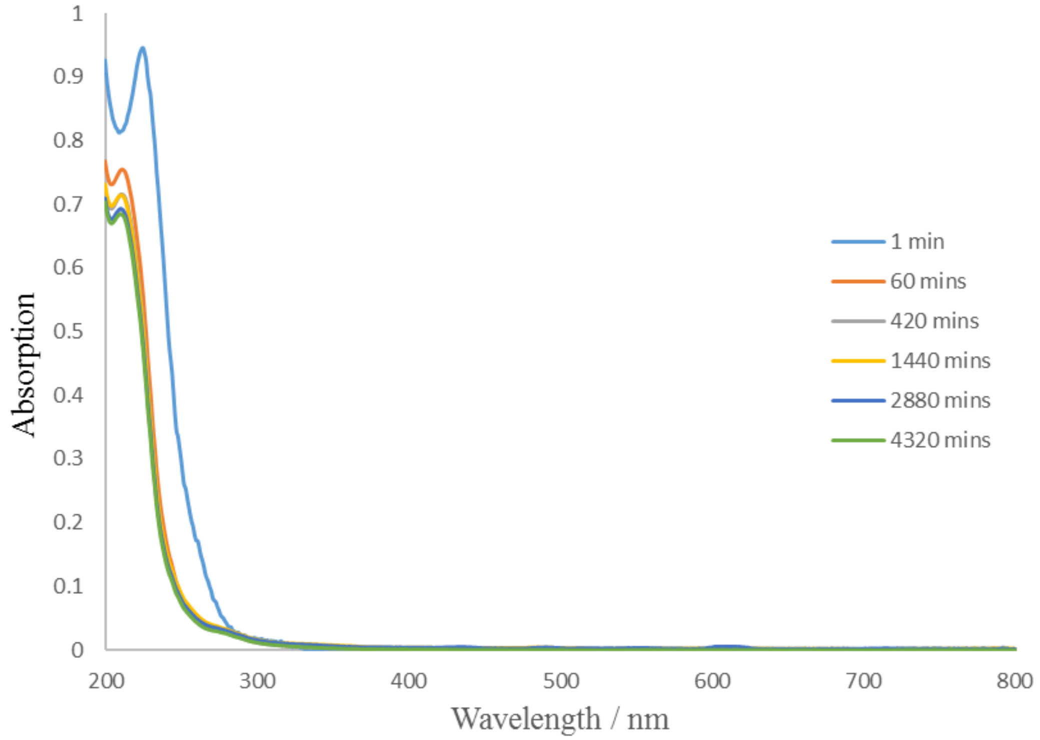

Figure S7: Stacked UV-Vis spectra of $Ag_8L_2(OAc)_4$ at pH 2 solution.

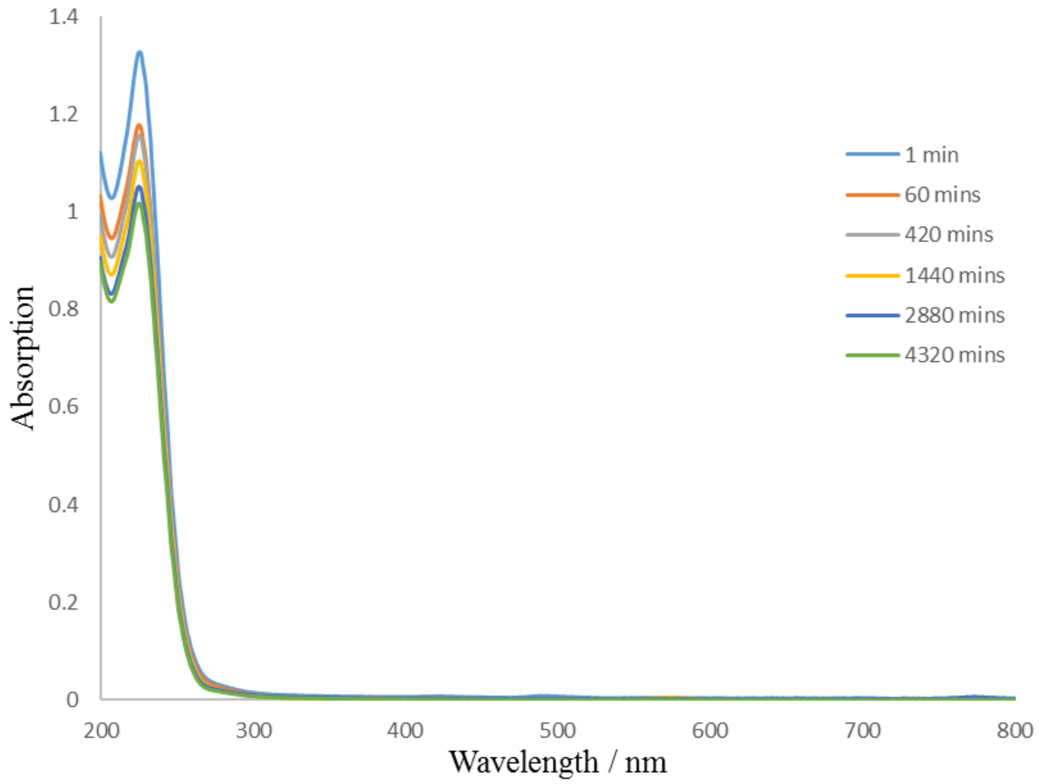

Figure S8: Stacked UV-Vis spectra of $Ag_8L_2(OAc)_4$ at pH 4 solution.

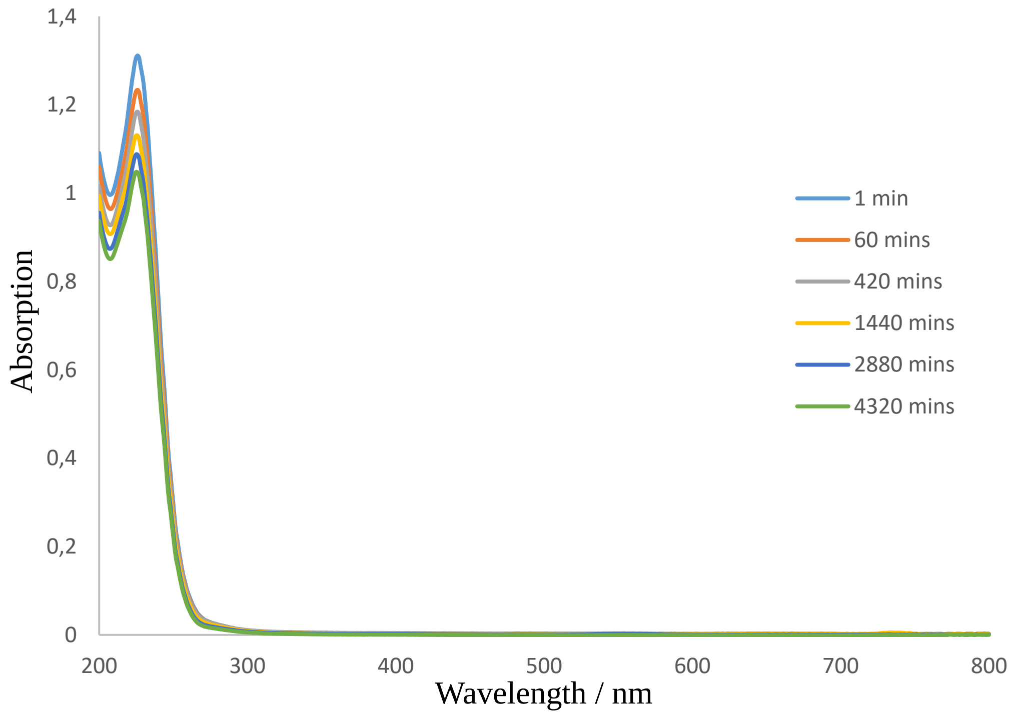

Figure S9: Stacked UV-Vis spectra of $Ag_8L_2(OAc)_4$ at pH 5 solution.

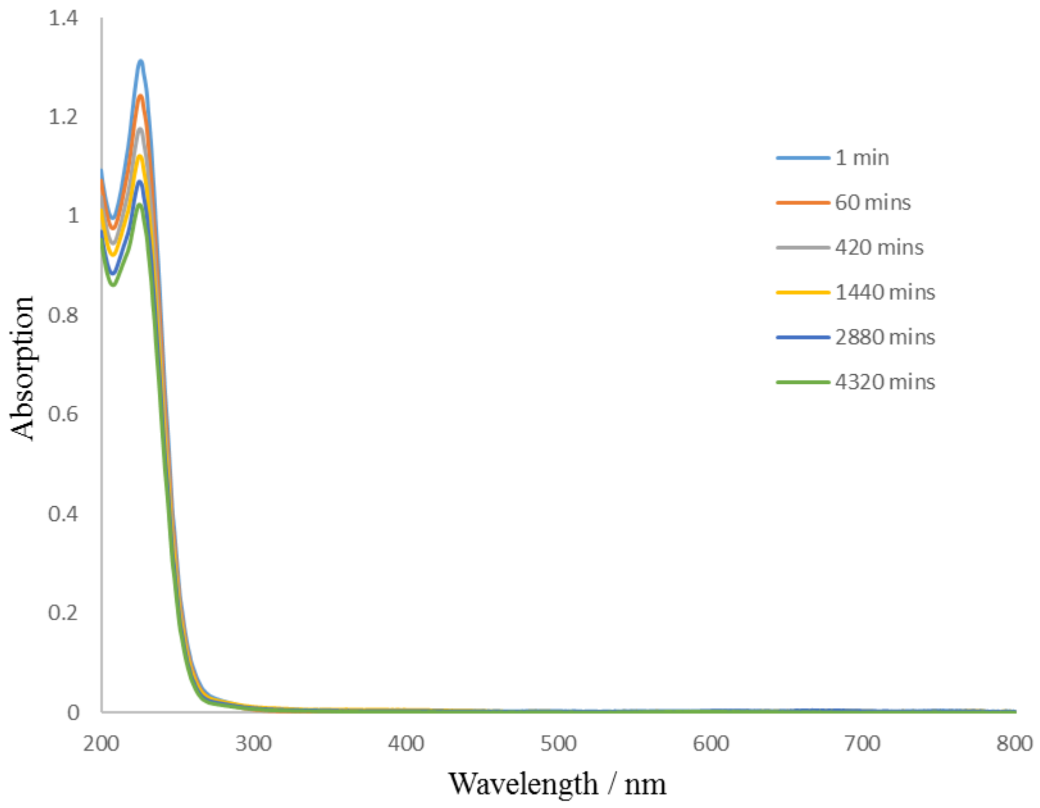

Figure S10: Stacked UV-Vis spectra of $Ag_8L_2(OAc)_4$ at pH 6 solution.

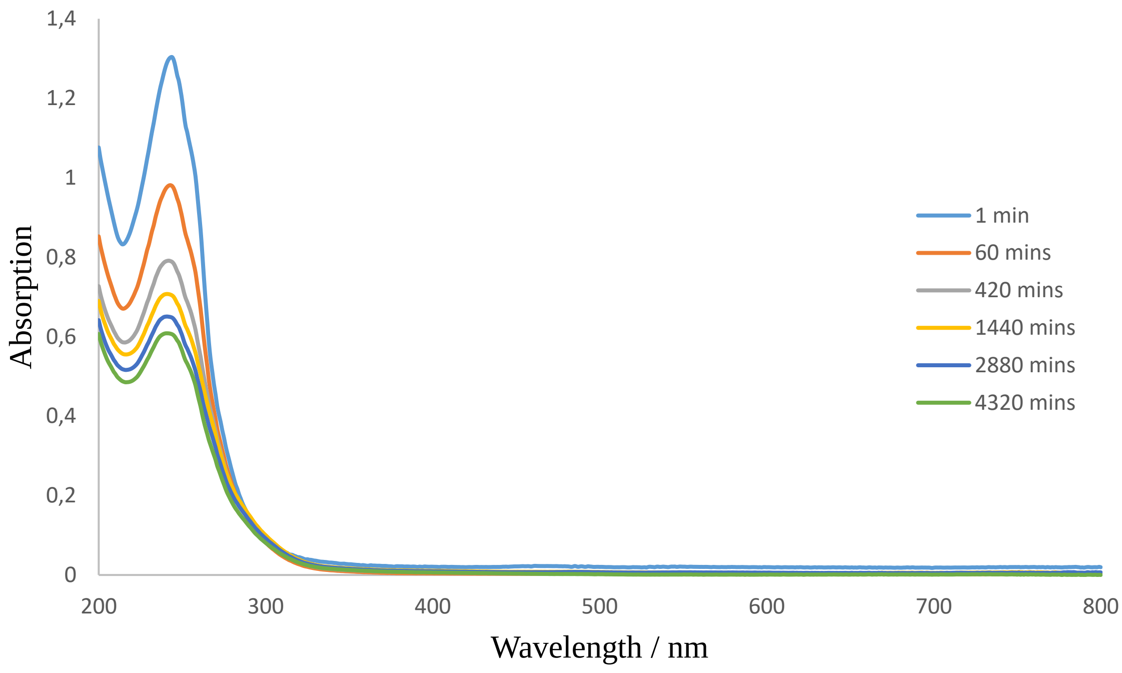

Figure S11: Stacked UV-Vis spectra of $Au_8L_2(OAc)_4$ at pH 2 solution

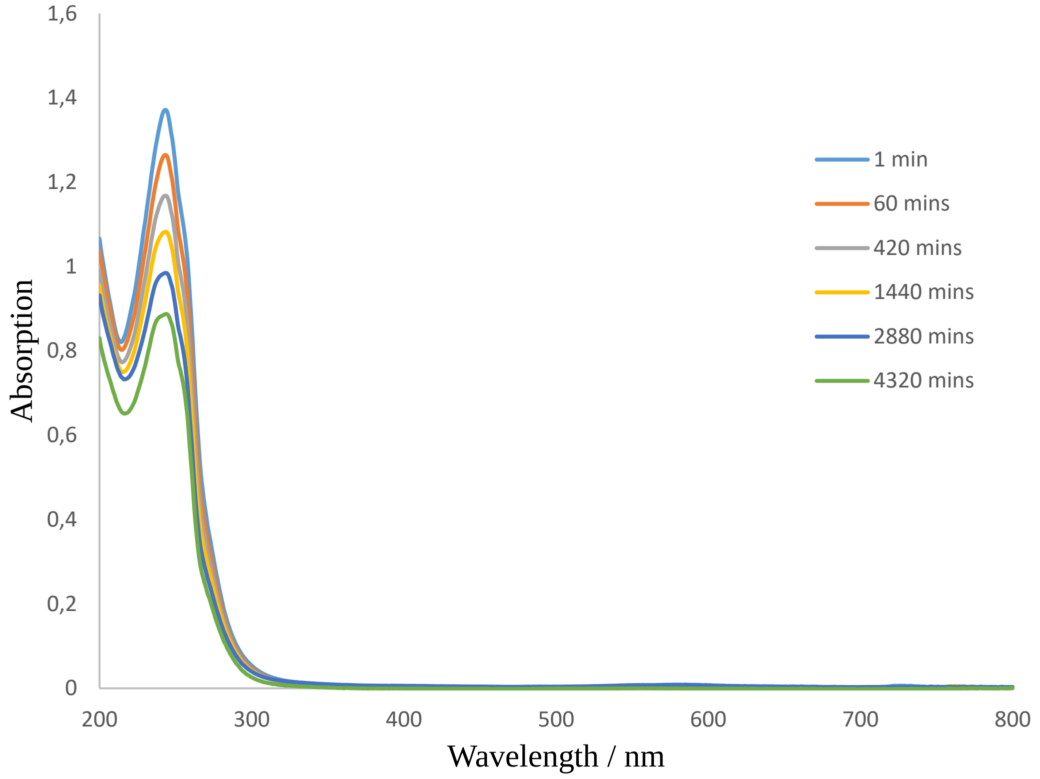

Figure S12: Stacked UV-Vis spectra of $Au_8L_2(OAc)_4$ at pH 4 solution

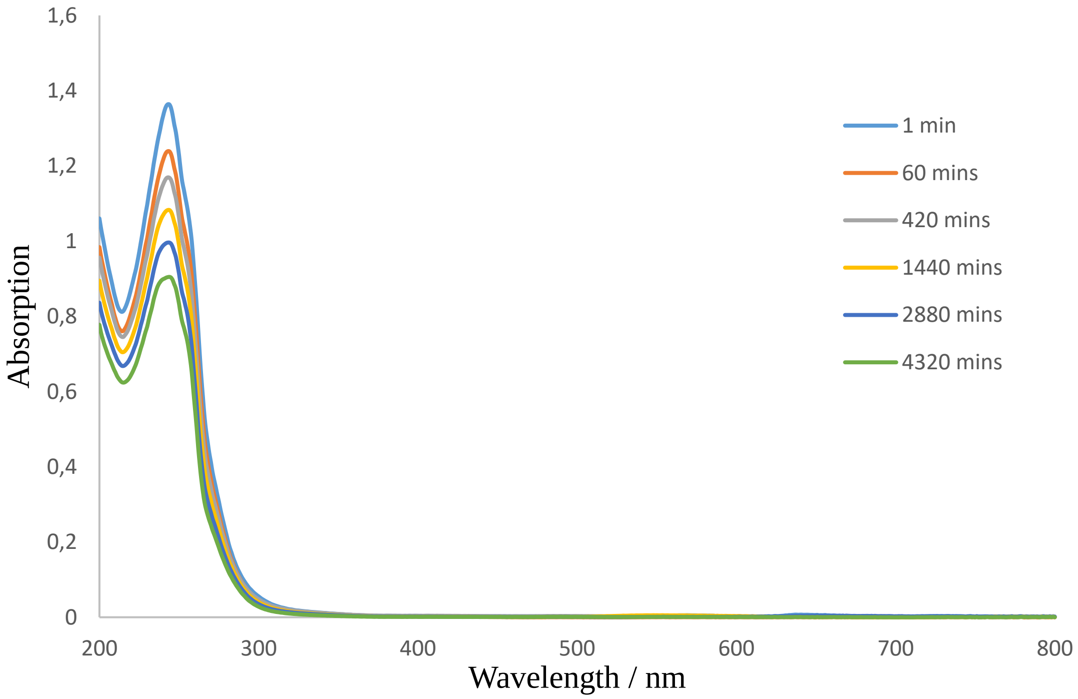

Figure S13: Stacked UV-Vis spectra of $Au_8L_2(OAc)_4$ at pH 5 solution

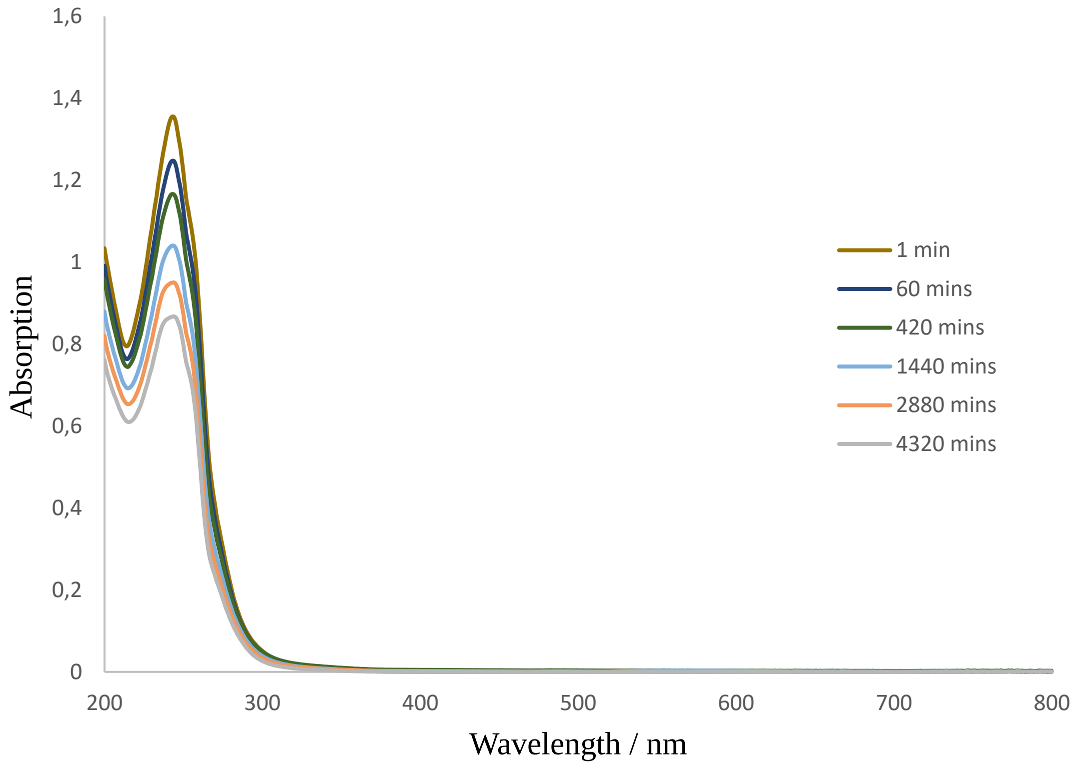

Figure S14: Stacked UV-Vis spectra of $Au_8L_2(OAc)_4$ at pH 6 solution

4 ^{1}H NMR spectrum of $[Au_8L_2](OAc)_4$ (6) in D_2O at pH 2

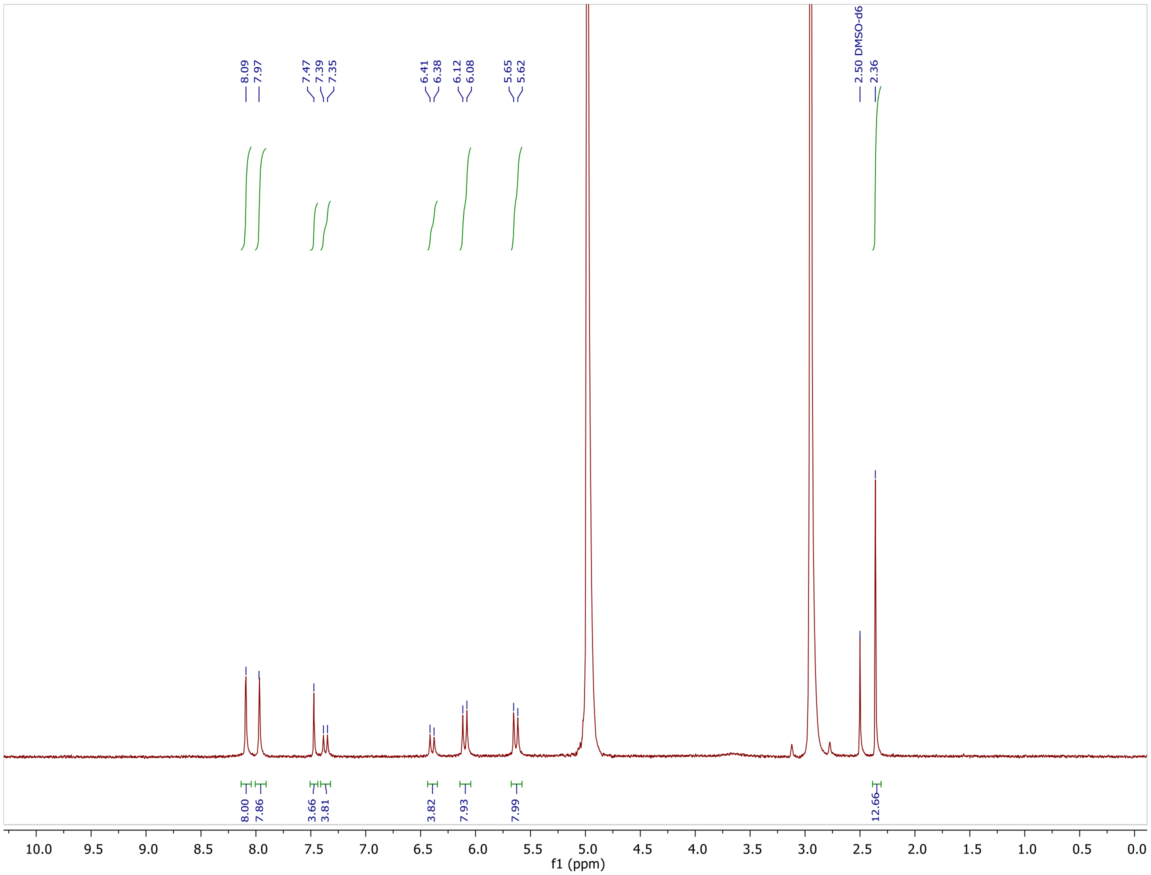

Figure S15: ^{1}H NMR (400 MHz, 298 K) of 1.7 mg $[Au_8L_2](OAc)_4$ in 0.3 ml D_2O into a 20 mM solution of HOTf in equal volume of D_2O. The proton concentration of resulting solution is 10 mM, corresponding to pH 2.
